# Pharmacological Inhibition of TFF3 Enhances Sensitivity of CMS4 Colorectal Carcinoma to 5-Fluorouracil through Inhibition of p44/42 MAPK

**DOI:** 10.3390/ijms20246215

**Published:** 2019-12-09

**Authors:** Ru-Mei Chen, Yi-Shiou Chiou, Qing-Yun Chong, Han-Ming Poh, Tuan-Zea Tan, Meng-Yi Zhang, Lan Ma, Tao Zhu, Vijay Pandey, Basappa, Alan Prem Kumar, Peter E. Lobie

**Affiliations:** 1 Cancer Science Institute of Singapore, National University of Singapore, Singapore 117599, Singapore; chenrumei@u.nus.edu (R.-M.C.); csicqy@nus.edu.sg (Q.-Y.C.); Han_ming89@hotmail.com (H.-M.P.); csittz@nus.edu.sg (T.-Z.T.); zhangmengyi@u.nus.edu (M.-Y.Z.); 2 Department of Pharmacology, Yong Loo Lin School of Medicine, National University of Singapore, Singapore 117600, Singapore; 3 Tsinghua Berkeley Shenzhen Institute (TBSI), Tsinghua University, Shenzhen 518055, Guangdong, China; chiouyishiou@gmail.com (Y.-S.C.); malan@sz.tsinghua.edu.cn (L.M.); vijaypandey7@hotmail.com (V.P.); 4 College of Pharmacy, State Key Laboratory of Medicinal Chemical Biology, Nankai University, Tianjin 300350, China; 5 Shenzhen Bay Laboratory, Shenzhen 518055, Guangdong, China; 6 Hefei National Laboratory for Physical Sciences at Microscale and School of Life Sciences, University of Science and Technology of China, Hefei 230027, Anhui, China; zhut@ustc.edu.cn; 7 Laboratory of Chemical Biology, Department of Studies in Organic Chemistry, University of Mysore, Manasagangotri, Mysore 570006, India; salundibasappa@gmail.com; 8 Cancer Program, Medical Science Cluster, Yong Loo Lin School of Medicine, National University of Singapore, Singapore 117599, Singapore

**Keywords:** trefoil factor 3, CMS4 CRC, ERK1/2, 5-FU, cancer stem cell, targeted therapy

## Abstract

Increased expression of trefoil factor 3 (TFF3) has been reported in colorectal carcinoma (CRC), being correlated with distant metastasis and poor clinical outcomes. Amongst the CRC subtypes, mesenchymal (CMS4) CRC is associated with the worst survival outcome. Herein, the functional roles of TFF3 and the pharmacological inhibition of TFF3 by a novel specific small molecule TFF3 inhibitor—2-amino-4-(4-(6-fluoro-5-methylpyridin-3-yl)phenyl)-5-oxo-4H,5H-pyrano[3,2-c]chromene-3-carbonitrile (AMPC) in CMS4 CRC was explored. Forced expression of TFF3 in CMS4 CRC cells promoted cell proliferation, cell survival, foci formation, invasion, migration, cancer stem cell like behaviour and growth in 3D Matrigel. In contrast, siRNA-mediated depletion of TFF3 or AMPC inhibition of TFF3 in CMS4 CRC cells decreased oncogenic behaviour as indicated by the above cell function assays. AMPC also inhibited tumour growth in vivo. The TFF3-stimulated oncogenic behaviour of CMS4 CRC cells was dependent on TFF3 activation of the p44/42 MAPK (ERK1/2) pathway. Furthermore, the forced expression of TFF3 decreased the sensitivity of CMS4 CRC cells to 5-fluorouracil (5-FU); while depleted TFF3 expression enhanced 5-FU sensitivity in CMS4 CRC cells. 5-FU treatment induced TFF3 expression in CMS4 CRC cells. AMPC, when used in combination with 5-FU in CMS4 CRC cells exhibited a synergistic inhibitory effect. In summary, this study provides functional evidence for TFF3 as a therapeutic target in CMS4 CRC.

## 1. Introduction

Approximately 15–25% of colorectal carcinoma (CRC) patients are diagnosed at the metastatic stage and the majority of these metastases are not resectable [1,2]. For these patients, chemotherapy, radiation and targeted therapies, will be used in an attempt to improve overall outcome. Although CRC is a heterogeneous disease, the standard of care for stage II/III CRC remains chemotherapy-containing regimens [3]. Currently, there is a lack of clinical stratification to guide treatment decisions for CRC [4]. A classification system based on clinical relevance for colorectal cancer is the consensus molecular subtype (CMS) system: microsatellite instability immune (CMS1), canonical (CMS2), metabolic (CMS3), or mesenchymal (CMS4) [4]. CMS subtype classification is considered as the most robust classification system currently available for colorectal cancer [4,5]. Studies have shown that subtype-specific therapies based on CMS classification can be used to predict therapeutic responses in the clinic and treatment responses varies between subtypes [6,7]. In comparison to the other CMS subtypes, CMS4 subtype patients tends to be diagnosed at a more advanced stage, resulting in a worse overall and relapse-free survival [4,8]. In terms of in vitro response to chemotherapy, CMS4 cell lines are more resistant to 5-FU–induced apoptosis compared with cells belonging to CMS subtypes 1–3 [9].

Trefoil factor 3 (TFF3) belongs to a family of trefoil factor proteins, characterised by the presence of the trefoil domain, which is a triple looped structure formed by three disulphide bonds [10,11]. TFF3 is predominantly secreted by the goblet cells of the intestines and apparently functions in epithelial restitution with anti-anoikis and motogenic functions [12]. Analyses of clinical samples have revealed an increased level of TFF3 protein in the serum of CRC patients [13]. TFF3 also likely functions in CRC metastasis, as increased expression of TFF3 mRNA, and protein has been observed in CRC metastatic to liver [14]. TFF3 forms a homodimer via the presence of a seventh cysteine (Cys57) residue near its carboxyl terminus [15]. TFF3 has also been reported to form heterodimers with other proteins; for example, the predominant form of TFF3 in intestinal epithelia is a heterodimer with FCGBP [16]. Numerous studies have reported differences in the functions of the monomeric and dimeric forms of TFF3, with homodimerization required for the pro-proliferative and anti-apoptotic functions of TFF3 [17,18]. Based on this knowledge, a small molecule inhibitor of TFF3 has been designed and designated as AMPC (2-amino-4-(4-(6-fluoro-5-methylpyridin-3-yl)phenyl)-5-oxo-4H,5H-pyrano[3,2-c]chromene-3-carbonitrile), which specifically targets the Cys57-Cys57 bond of dimeric TFF3, resulting in the monomerization of dimeric TFF3 to inhibit the pro-proliferative and anti-apoptotic functions of homodimeric TFF3 [19].

In this study, we demonstrated the oncogenic potential of TFF3 in CMS4 CRC, which supports the potential of TFF3 as a diagnostic marker and therapeutic target in this subtype of CRC. The novel small molecule TFF3 inhibitor, AMPC, could potentially be utilized as a single agent or in combination with 5-FU to improve current clinical treatment in TFF3 positive CMS4 CRC.

## 2. Results

### 2.1. Forced Expression of TFF3 Promotes Oncogenic Behaviour of CMS4 CRC Cells in Vitro

TFF3 expression has been reported as an independent prognostic factor for CRC and also predicted early recurrence of CRC [20]. Furthermore, the expression of TFF3 has been reported to be increased by chemo-radiotherapy, resulting in reduced sensitivity to treatment and higher risk of relapse in rectal cancer [21]. To determine an association of TFF3 expression with patient survival outcomes in the CRC CMS subtypes, the available data in TCGA was therefore analysed. The TCGA cohort demonstrated that patients within the CMS4 subtype with low tumour expression of TFF3 exhibited a greater 10-year overall survival outcome compared to patients with high tumour expression of TFF3 (Figure S1A). The expression of TFF3 in a panel of CMS4 CRC cell lines was therefore determined by qPCR and western blot analysis (Figure S2A,B). Amongst this cell line panel, a pair of cell lines (SW480 and SW620) were derived from the same patient. SW480 cells were derived from the primary tumour while SW620 cells were derived from a lymph node metastasis [22]. The high expression of TFF3 in SW620 cells but not in SW480 cells (Figure S2A,B) has been previously reported [13]. For this study, Caco2 cells with moderate TFF3 expression and SW620 cells with high TFF3 expression were chosen to establish forced expression and depletion of TFF3 cell line models respectively.

**Figure 1:**
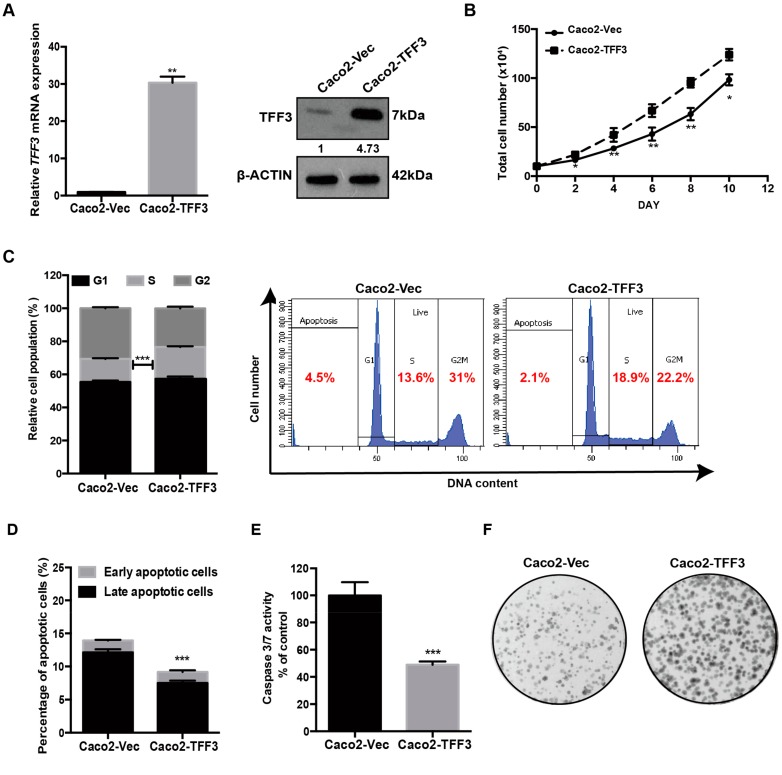

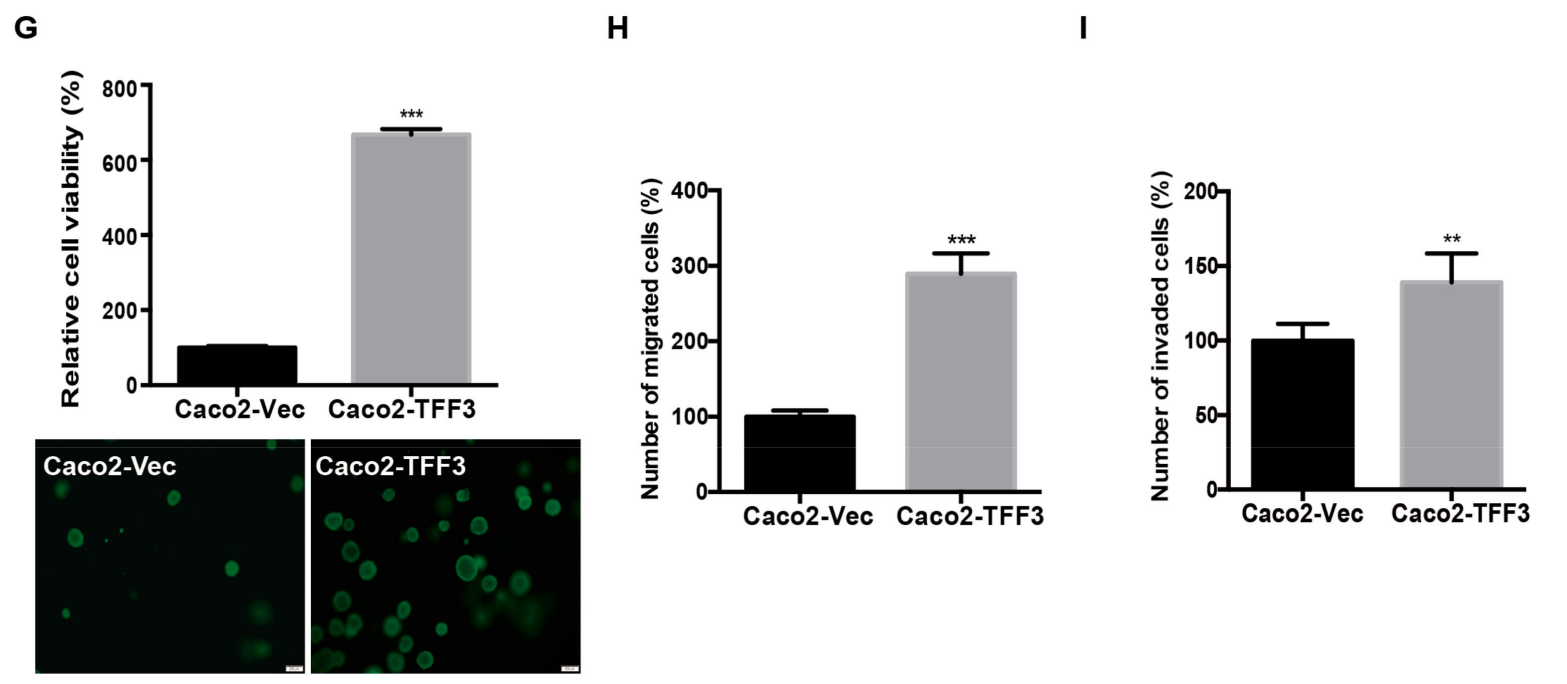
Forced expression of trefoil factor 3 (TFF3) promotes oncogenic behaviour of Caco2 cells. (**A**) Detection of TFF3 expression with qPCR and western blot. β-ACTIN was used as input control. (**B**) Total cell count. On day 0, 10 × 10^4^ cells/well were seeded in six-well plates in triplicate. Cell numbers were counted at the indicated time points. (**C**) Cell cycle progression of cells cultured in 2% FBS medium was determined using propidium iodide (PI) staining followed by FACS analysis. The percentages of cells in each cell cycle phase are plotted. (**D**) Annexin-V/PI apoptotic cell death was determined after 24 h serum deprivation. The percentages of early apoptotic (Annexin-V-positive/PI-negative) and late apoptotic (Annexin-V-positive/PI-positive) cells are plotted. (**E**) Caspase 3/7 activities in the cells were determined after 24 h serum deprivation. (**F**) Foci formation. Cells were seeded in six-well plates and cultured for 10 days prior to fixation and crystal violet staining. (**G**) Three-dimensional Matrigel growth. Cells were cultured in 5% FBS medium containing 4% Matrigel. Cell viability was determined by AlamarBlue assay after nine days. Fold change of cell viability relative to –Vec cells is shown in the histogram. Representative microscopic images of viable colonies formed by the respective cells in 3D matrigel and stained by CellTrace calcein green acetoxymethyl (AM) are shown. Scale bar: 200 μm. (**H**) Cell migration assay. The cells that migrated across the transwell membrane after 12 h were stained with Hoechst 33342 and counted under the fluorescence microscope. Fold change of migrated cells relative to –Vec cells is shown in the histogram. (**I**) Cell invasion assay. Cells that invaded across the 10% Matrigel-coated transwell membrane after 24h were stained with Hoechst 33342 and counted under the fluorescence microscope. Fold change of invaded cells relative to –Vec cells is shown in the histogram. Data are expressed as mean ±SD. **, *p* < 0.01; ***, *p* < 0.001.

To determine the effects of TFF3 on CMS4 CRC progression, a forced expression model was established in Caco2 cells by stable transfection with TFF3 expression plasmid pIRESneo3-TFF3 (designated as Caco2-TFF3) or with empty vector pIRESneo3 (designated as Caco2-vec). The forced-expression of TFF3 in stably transfected Caco2 cells was verified at both mRNA and protein levels (Figure 1A). The Caco2-TFF3 cells exhibited a significantly greater increase in total cell number than the Caco2-Vec control cells over a period of 10 days (Figure 1B). Cell cycle analysis showed that Caco2-TFF3 cells exhibited a 6.3% increase in the S-phase fraction as compared to Caco2-Vec cells (Figure 1C). Furthermore, Annexin V/PI apoptosis assays revealed that forced expression of TFF3 significantly decreased apoptosis of Caco2 cells induced by serum deprivation (Figure 1D). Caspase-3/7 activity was consistently decreased in Caco2-TFF3 compared with Caco2-Vec cells after serum deprivation (Figure 1E). As shown in Figure 1F, Caco2-TFF3 cells also formed more and larger foci as compared to Caco2-Vec cells. We also determined the growth of Caco2 cells in 3D Matrigel, which better approximates in vivo growth conditions. Caco2-TFF3 cells formed more and larger-sized colonies in 3D Matrigel, with significantly higher cell viability, as compared to Caco2-Vec cells (Figure 1G). Transwell migration and invasion assays were used to measure the migration and invasion capacity of Caco2 cells with forced expression of TFF3. As shown in Figure 1H,I, Caco2 cells with forced expression of TFF3 exhibited significantly increased capacities for cell migration and invasion as compared to the vector control cells. Hence, forced expression of TFF3 promoted the proliferation, survival, migration and invasion of CMS4 CRC cells.

### 2.2. Depleted Expression of TFF3 Decreases Oncogenic Behaviour of CMS4 CRC Cells in Vitro

**Figure 2:**
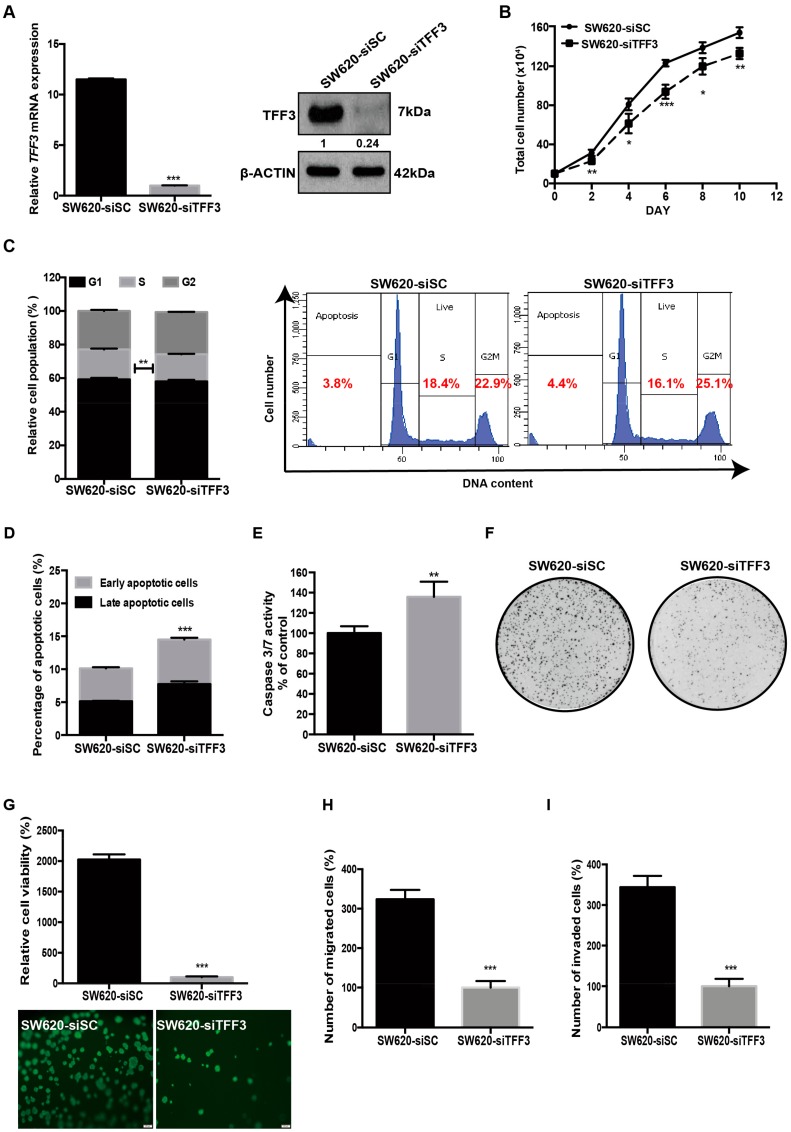
Depleted expression of TFF3 decreases oncogenic behaviour in SW620 cells. SW620 cells were transiently transfected with TFF3 siRNA (designated SW620-siTFF3) or scrambled siRNA (SW620-siSC). (**A**) Detection of TFF3 expression by qPCR and Western blot analysis. β-ACTIN was used as input control. (**B**) Total cell count. Cells were seeded in six-well plates in triplicate at 10 × 10^4^ cells/well on day 0. Cell numbers were counted at the indicated time points. (**C**) Cell cycle progression of cells cultured in 2% FBS medium was determined using PI staining followed by FACS analysis. The percentages of cells in each cell cycle phase are plotted. (**D**) Annexin-V/PI apoptotic cell death was determined after 24 h serum deprivation. The percentages of early apoptotic (Annexin-V-positive/PI-negative) and late apoptotic (Annexin-V-positive/PI-positive) cells are plotted. (**E**) Caspase 3/7 activities in the cells were determined after 24 h serum deprivation. (**F**) Foci formation. Cells were seeded in six-well plates and cultured for 10 days prior to fixation and crystal violet staining. (**G**) 3D Matrigel growth. Cells were cultured in 5% FBS medium containing 4% Matrigel. Cell viability was determined by AlamarBlue assay after eight days. Fold change of cell viability relative to –Vec cells is shown in the histogram. Representative microscopic images of viable colonies formed by the respective cells in 3D Matrigel and stained by CellTrace Calcein Green AM are shown. Scale bar: 200 μm. (**H**) Cell migration assay. Cells that migrated across the Transwell membrane after 12h were stained with Hoechst 33342 and counted under the fluorescence microscope. Fold change of migrated cells relative to –Vec cells is shown in the histogram. (**I**) Cell invasion assay. Cells that invaded across the 10% Matrigel-coated transwell membrane after 24 h were stained with Hoechst 33342 and counted under the fluorescence microscope. Fold change of invaded cells relative to –Vec cells is shown in the histogram. Data are expressed as mean ±SD. **, *p* < 0.01; ***, *p* < 0.001.

Depletion of TFF3 in SW620 cells was achieved by transient transfection with siRNA targeting TFF3 mRNA (designated as SW620-siTFF3) or scrambled siRNA (siSC) (designated as SW620-siSC) as negative control. The depletion of TFF3 mRNA and protein levels in SW620 cells was confirmed by real-time PCR and western blot analysis (Figure 2A). In contrast with the forced expression of TFF3, the total cell number was decreased with depletion of TFF3 in SW620 over a 10-day culture period (Figure 2B). Depletion of TFF3 in SW620 also produced a decrease in the S-phase fraction (Figure 2C). In addition, siRNA-mediated TFF3 depletion in SW620 significantly increased apoptotic cell death upon serum deprivation (Figure 2D). Consistently, SW620-siTFF3 cells exhibited higher caspase-3/7 activity than SW620-siSC cells in serum-deprived conditions (Figure 2E). Foci formation revealed fewer and smaller colonies formed by SW620-siTFF3 cells compared with SW620-siSC cells (Figure 2F). There was also a significant decrease in cell viability of SW620-siTFF3 cells in 3D Matrigel as compared to SW620-siSC cells (Figure 2G). TFF3-depleted SW620 cells also exhibited a reduction in both cell migration and cell invasion capacities as compared to the –Vec cells (Figure 2H,I).

### 2.3. TFF3 Promotes CSC-Like Properties in CMS4 CRC Cells

**Figure 3:**
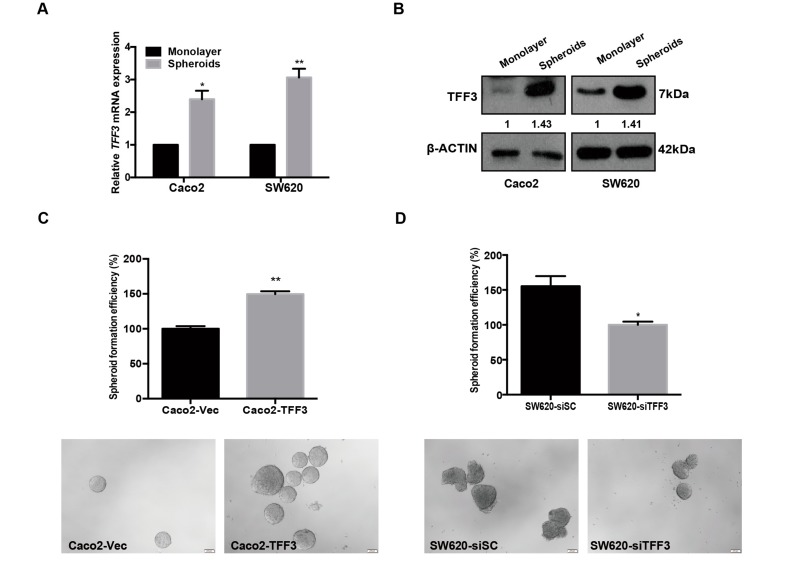

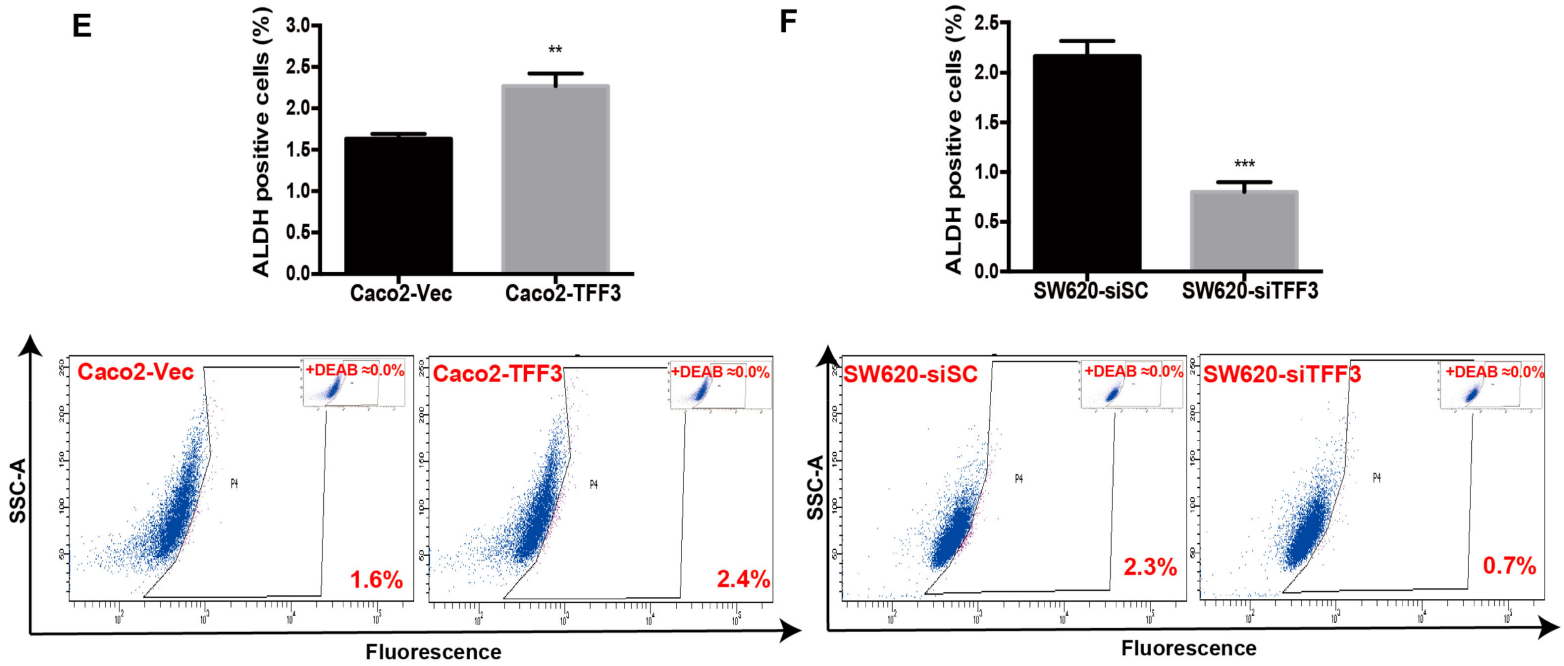
TFF3 promoted cancer stem cell–like behaviour in mesenchymal colorectal carcinoma (CMS4 CRC) cells. (**A**) qPCR analysis of TFF3 mRNA in Caco2 and SW620 cells was normalized to *β-ACTIN*. TFF3 expression in monolayer vs. spheroid culture is presented as fold change. (**B**) Western blot analysis for protein expression of TFF3 in monolayer or spheroid culture. β-ACTIN was used as input control. (**C**) Spheroid formation assay. Caco2-Vec and Caco2-TFF3 cells were seeded in ultra-low attachment plates in spheroid growth media for 10 days. Results are presented as percentages relative to the respective control cells (numbers of colonizes are shown). Representative images are shown. Scale bar: 200 μm. (**D**) Spheroid formation of SW620-siSC and SW620-siTFF3 cells as in (**C**). (**E**) The ALDH1+ cell population was determined in Caco2 cells using the ALDEFLUOR assay. Cells were incubated with Aldefluor substrate (BAAA, BoDIPY^®^-aminoacetaldehyde) to define ALDH1 positivity and diethylaminobenzaldehyde (DEAB), a specific inhibitor of ALDH1, was used as control to establish the baseline fluorescence. The percentage of the ALDH1+ cells are plotted. (**F**) The ALDH1+ cell population was determined in SW620-siSC and SW620-siTFF3 cells using ALDEFLUOR assay as in (**E**). Data are expressed as mean ±SD. *, *p* < 0.05; **, *p* < 0.01; ***, *p* < 0.001.

Cancer stem cell (CSC)-like properties have been postulated to drive chemoresistance and metastasis resulting in poor clinical outcomes [23,24]. Gene ontology analysis revealed that the CMS4 subtype is significantly enriched for the embryonic CSC-like signature as compared to CMS1-3 subtype in clinical cohorts (Figure S1B). To examine the potential function of TFF3 in promoting CSC-like behaviour in CRC cells, wild type Caco2 and SW620 cells were grown in parallel under normal monolayer culture condition or in serum-free defined media under ultra-low attachment condition to form colonospheres, which are enriched for a CSC-like population [25]. As shown in Figure 3A, the mRNA expression of TFF3 was increased 2–3 fold in spheroid culture in comparison to cells in monolayer culture. The protein expression of TFF3 was also observed to be higher in spheroid compared to monolayer culture (Figure 3B). This data is consistent with previous findings that TFF3 promotes CSC-like behaviour in trastuzumab resistant mammary carcinoma cells and hepatocellular carcinoma cells [26,27].

Caco2 cells with forced expression of TFF3 exhibited significantly increased size and number of spheroids when compared with –Vec cells (Figure 3C). In contrast, depletion of TFF3 in SW620 cells significantly decreased spheroid formation (Figure 3D). As aldehyde dehydrogenase-1 (ALDH1) is known as a cancer stem cell marker [28] the effect of TFF3 on modulating the ALDH1-positive cell population was examined. Results of flow cytometry analysis revealed that forced expression of TFF3 significantly increased the percentage of ALDH1-positive cells in Caco2 cells (Figure 3E), while the depletion of TFF3 significantly decreased the ALDH1-positive population in SW620 cells (Figure 3F). Such observations indicate that TFF3 increases the CSC-like population in CMS4 CRC cells.

### 2.4. Pharmacological Inhibition of TFF3 Decreases Oncogenicity of CMS4 CRC Cells

**Figure 4:**
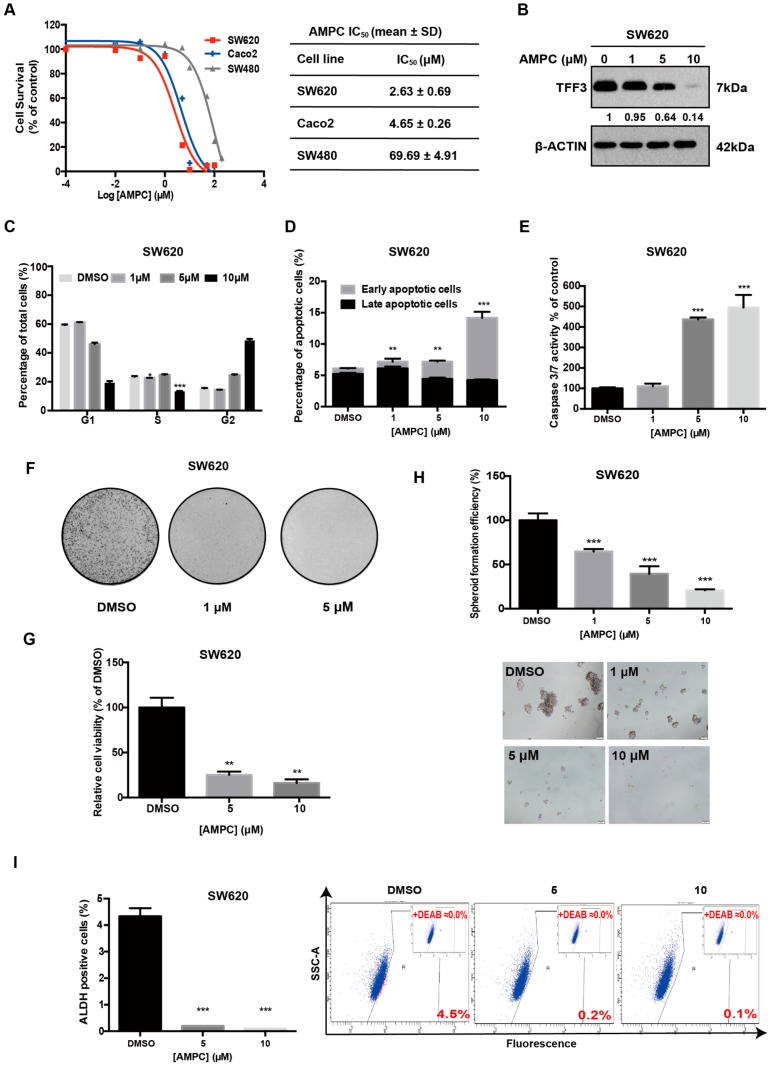
Pharmacological inhibition of TFF3 by 2-amino-4-(4-(6-fluoro-5-methylpyridin-3-yl)phenyl) -5-oxo-4H,5H-pyrano[3,2-c]chromene-3-carbonitrile (AMPC) decreases oncogenicity of SW620 cells. (**A**) IC_50_ values of AMPC in SW620, Caco2, and SW480 cells. (**B**) TFF3 protein levels after 24 h AMPC treatment were determined by Western blot analysis. β-ACTIN was used as input control. (**C**) Cell cycle analysis after 24 h AMPC treatment. The percentages of cells in each cell cycle phase are plotted and statistical significance in the difference in the percentages of AMPC- and vehicle DMSO–treated cells in the S phase is shown. (**D**) Annexin-V/PI apoptotic cell death was determined after 24 h AMPC treatment. The percentages of early apoptotic and late apoptotic cells are plotted and the statistical significance in the difference in the percentages of apoptotic cells between AMPC- and vehicle DMSO–treated cells is shown. (**E**) Caspase 3/7 activities were determined after 24 h AMPC treatment. (**F**) Foci formation. Cells were seeded in six-well plates and treated with the indicated concentrations of AMPC for a period of 12 days. The resulting foci formed were fixed and stained with crystal violet. (**G**) Three-dimensional Matrigel growth. Cells were cultured in 5% FBS medium containing 4% Matrigel for three days prior to treatment with AMPC for six days. The fold change in cell viability after AMPC treatment is shown in the histogram. (**H**) Spheroid formation assay. Cells were seeded in ultra-low attachment plates in spheroid growth media and treated with the indicated concentrations of AMPC. After 14 days, spheroid growth was measured by AlamarBlue. The fold change in cell viability after AMPC treatment is shown in the histogram. Scale bar: 200 μm. (**I**) ALDEFLUOR assay. SW620 cells were treated with AMPC for 24h. The cells were incubated with ALDEFLOUR substrate to define ALDH1-positive population while a specific inhibitor of ALDH1 (DEAB) was used to establish baseline fluorescence. The percentages of ALDH1-positive cells after AMPC treatment were determined by flow cytometry. Data are expressed as mean ±SD. **, *p* < 0.01; ***, *p* < 0.001.

A novel small molecule TFF3 inhibitor, AMPC, has been developed using a structure-based approach and high-throughput virtual screening [19]. AMPC utilizes cysteine 57 essential for the homodimerization of TFF3 and therefore disrupts dimeric TFF3 function which includes the promotion of cell survival and proliferation [29]. We therefore utilized AMPC to examine the effects of pharmacological inhibition of TFF3 in CMS4 CRC cells. The IC_50_ values of AMPC in CMS4 CRC cells were determined (Figure 4A). We observed that SW620 cells with high TFF3 expression were slightly more sensitive to AMPC than Caco2 cells with low TFF3 expression. SW480 cells, which lack significant TFF3 expression, also exhibited an IC_50_ at least a log order higher than SW620. Furthermore, siRNA-mediated depletion of TFF3 in SW620 cells abrogated the inhibitory effect of AMPC on cell viability (Figure S3A). Hence, these observations support the specificity of AMPC to TFF3.

As expected [19,26], AMPC treatment significantly reduced the total cellular levels of TFF3 in SW620 and Caco2 cells in a dose dependent manner (Figure 4B and Figure S3B). Consistent with the effect of siRNA-mediated depletion of TFF3, inhibition of TFF3 by AMPC decreased cell proliferation in CMS4 CRC cells, as shown by decrease in the S-phase population compared with dimethyl sulfoxide (DMSO)-treated control cells (Figure 4C and Figure S3C). Furthermore, the inhibition of TFF3 by AMPC in CMS4 CRC cells induced apoptosis in a dose dependent manner (Figure 4D and Figure S3D). Similarly, AMPC treatment increased caspase3/7 activity in CMS4 CRC cells (Figure 4E and Figure S3E). In addition, AMPC treatment reduced the ability of CMS4 CRC cells to form foci in a dose-dependent manner (Figure 4F and Figure S3F). Moreover, AMPC treatment suppressed colony growth of CMS4 CRC cells in 3D Matrigel (Figure 4G and Figure S3G). AMPC decreased the CSC-like property of CMS4 CRC cells indicated by reduced formation of colonospheres in a dose-dependent manner (Figure 4H and Figure S3H). AMPC also significantly decreased the ALDH1-positive cell population in CMS4 CRC cells in a dose dependent manner as compared with their respective DMSO-treated controls (Figure 4I and Figure S3I). In summary, AMPC is a potent and effective inhibitor of cell proliferation, survival, oncogenicity, 3D-growth, and CSC-like behaviour in TFF3-positive CMS4 CRC cells.

### 2.5. AMPC Suppresses SW620 Tumour Growth in a Mouse Xenograft Model 

**Figure 5:**
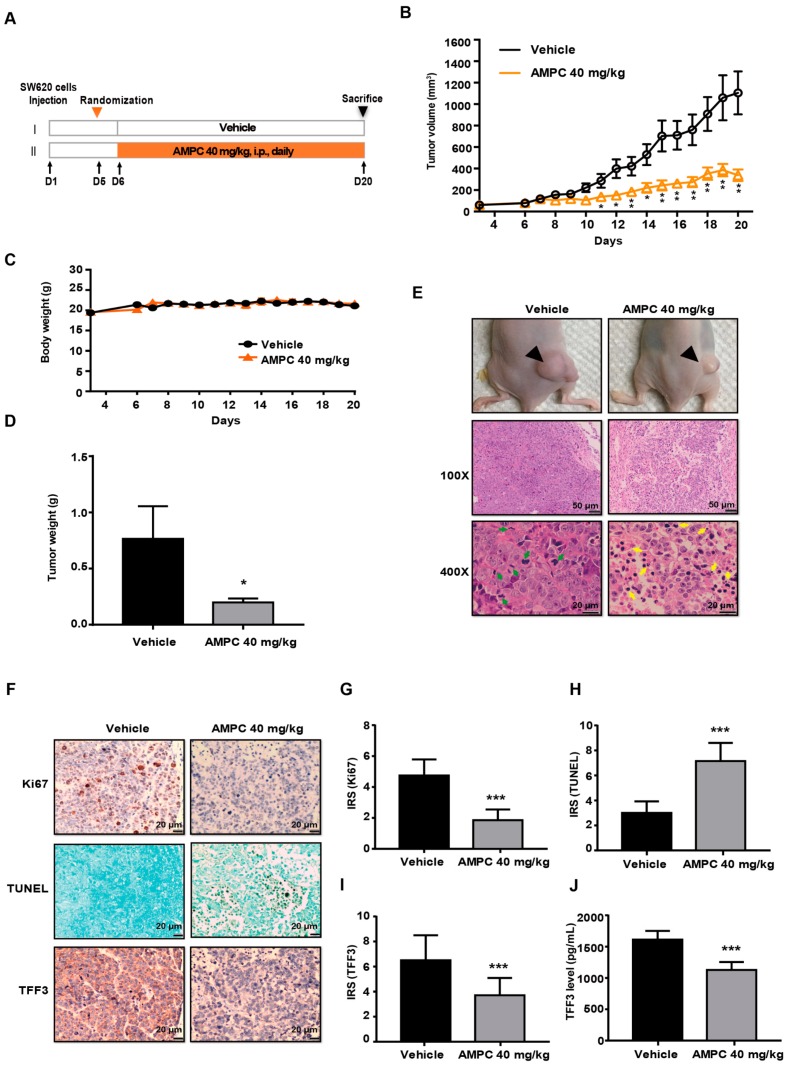
Inhibition of TFF3 by AMPC inhibits tumour growth in SW620 xenografts. (**A**) Schematic of experimental protocol described in Materials and Methods. BALB/c nude mice with SW620 cells–derived tumours were injected with AMPC (40 mg/kg/) or vehicle. (**B**) Tumour volumes and (**C**) body weights of mice were measured after tumour cell injection. (**D**) Tumour weights measured on termination of the experiment. Each bar represents the mean ± SD of eight mice. (**E**) Representative photographs of tumour sites (top) from a mouse 20 days after subcutaneous injection of SW620 cells and Haematoxylin and Eosin (H&E)-stained sections (bottom) of tumour (100× magnification, Scale bar: 50 μm; 400× magnification, Scale bar: 20 μm). (**F**) Immunohistochemical (IHC) staining of TFF3, Ki67, and TUNEL in tumours (200 × magnification, Scale bar: 20 μm). (**G**–**I**) Quantification of the immunohistochemical staining in (**F**). Each bar represents the mean ± SD of eight mice. (**J**) Serum levels of TFF3 were determined by ELISA. Results were statistically analyzed with one-way ANOVA followed by Tukey’s post hoc test. Statistical significance, * *p* < 0.05 and ** *p* < 0.01, *** *p* < 0.001, compared with the vehicle-treated group.

We next investigated the potential therapeutic effects of AMPC as a TFF3 inhibitor in vivo. SW620 cells were subcutaneously injected into nude mice. After tumour formation at six days, treatment was initiated (Figure 5A). A significant reduction of tumour volume was observed in AMPC-treated mice as compared to vehicle-treated mice from day 11 onward (Figure 5B). The difference in body weight between groups was not statistically significant indicating that drug treatment was well tolerated (Figure 5C). At the end of day 21, animals were sacrificed, and tumours were harvested for further analysis. The average tumour weight of the AMPC-treated group was significantly less than that of the vehicle-treated group (Figure 5D). As shown in Figure 5E, AMPC treatment resulted in larger areas of tumour necrosis and increased area of cells with apoptotic features. Furthermore, a marked reduction of Ki67 positive cells and an increase in TUNEL-positive apoptotic cells were also observed in SW620 tumours after two weeks of AMPC treatment (Figure 5F–H). Consistent with in vitro results, AMPC treatment significantly reduced tumour and serum TFF3 levels (Figure 5F,I,J), indicating that serum TFF3 could be used as a response marker to monitor the efficacy of AMPC treatment. 

### 2.6. TFF3 Activates the p44/42 MAPK (ERK1/2) Pathway in CMS4 CRC Cells

**Figure 6:**
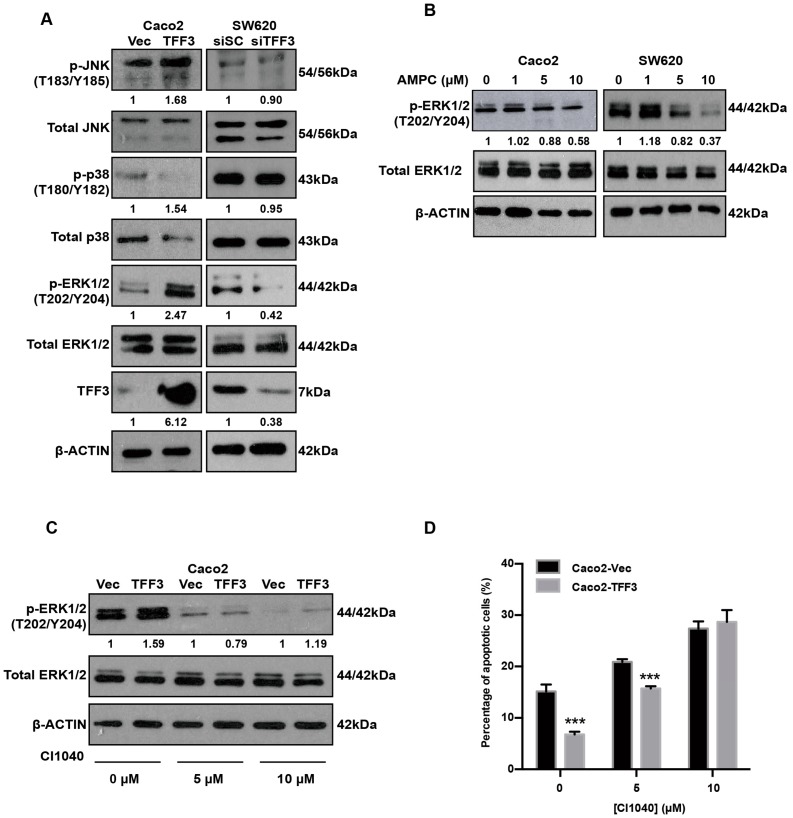
TFF3 activates the ERK1/2 (p44/42 MAPK) pathway in CMS4 CRC cells. (**A**) Western blot analysis of phosphorylated and total MAPKs in Caco2-Vec and Caco2-TFF3 cells and SW620-siSC and SW620-siTFF3 cells. β-ACTIN was used as input control. (**B**) Caco2 and SW620 cells were treated with the indicated concentrations of AMPC (with DMSO as vehicle) for 24 h. Levels of p-ERK1/2 and expression of total ERK1/2 were detected by western blot analysis. β-ACTIN was used as an input control. (**C**) Caco2-Vec and Caco2-TFF3 cells were treated with the indicated concentrations of MEK inhibitor (CI1040) or DMSO (vehicle control) for 24 h. Levels of p-ERK1/2 and expression of ERK1/2 were detected by western blot analysis. β-ACTIN was used as an input control. (**D**) Annexin V/PI staining analysis was performed to determine apoptosis in Caco2 stable cells. ***, *p* < 0.001.

TFF3 is known to activate HER1/2 [26] and trigger the activation of multiple downstream signal transduction molecules, including the MAPKs, PI3K, COX-2, and STAT3 [15,30]. We performed western blot analysis to examine the phosphorylation of MAPKs, namely JNK, p38, and ERK1/2. In Caco2 cells, an enhancement in ERK1/2 phosphorylation was observed with forced expression of TFF3 (Figure 6A). In contrast, with the depletion of endogenous TFF3 in SW620 cells, ERK1/2 phosphorylation was reduced, whereas the levels of total ERK1/2 remained unchanged (Figure 6A). However, modulation of JNK and p38 phosphorylation was not observed in Caco2 with forced expression of TFF3 nor in SW620 cells with depletion of TFF3 (Figure 6A). AMPC-mediated inhibition of TFF3 decreased activation of ERK1/2 in a dose-dependent manner (Figure 6B). To determine if ERK1/2 mediated TFF3 cellular functions in CMS4 CRC cells, a MEK inhibitor (CI-1040) was used that specifically inhibited the phosphorylation of ERK1/2 (Figure 6C). TFF3 enhancement of cell survival was reduced by 5 μM CI-1040 in Caco2-TFF3 cells (Figure 6D). Hence, TFF3 decreased apoptosis via activation of the ERK1/2 pathway in CMS4 CRC cells.

### 2.7. TFF3 Decreases 5-FU Sensitivity and 5-FU Combines with AMPC in a Synergistic Manner in CMS4 CRC Cells

**Figure 7:**
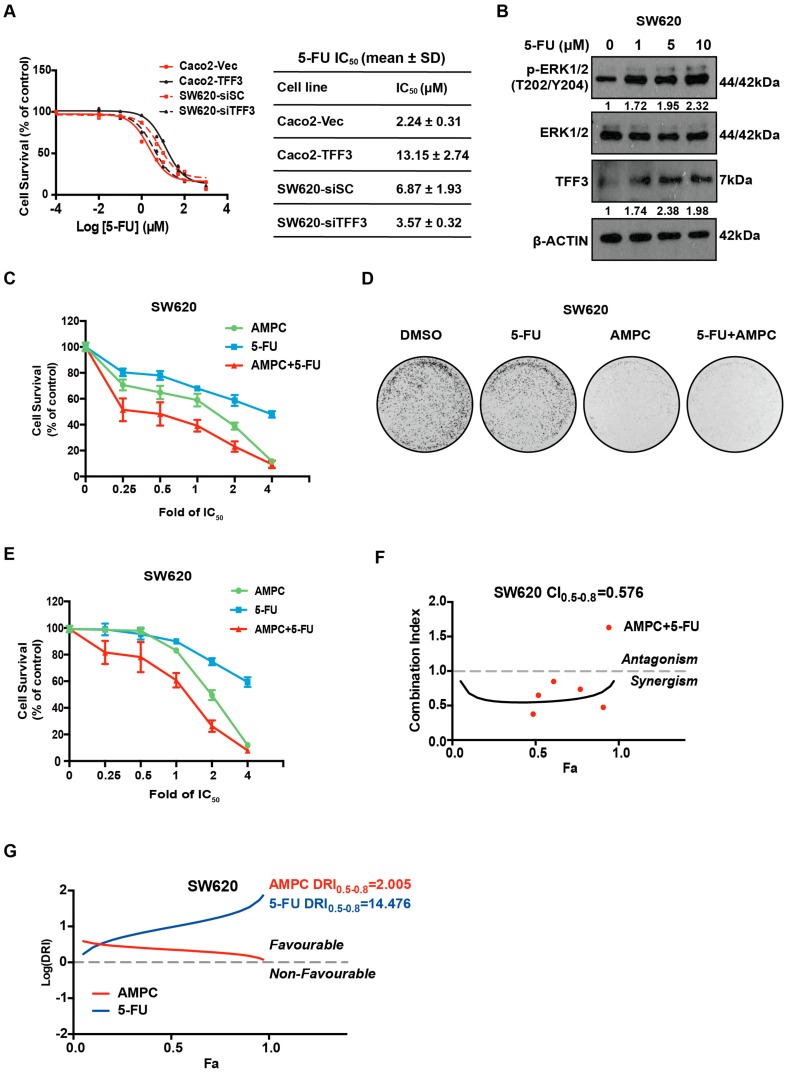
Synergistic inhibitory effects of AMPC and 5-Fluorouracil (5-FU) combination in SW620 cells. (**A**) The IC_50_ values of 5-FU in Caco2-Vec and Caco2-TFF3 cells and SW620-siSC and SW620-siTFF3 cells. Cells were seeded in 96-well plates and treated with the indicated concentrations of 5-FU. After 72 h, cell viability was measured by AlamarBlue assay. 5-FU IC_50_ values were calculated by the Graphpad Prism 5. (**B**) Western blot analysis. SW620 cells were treated with the indicated concentrations of 5-FU for 24 h. Levels of p-ERK1/2 and expression ofERK1/2 and TFF3 were determined by western blot analysis. β-ACTIN was used as an input control. (**C**) Cell viability assay for the drug combination of AMPC and 5-FU in SW620 cells. Cells were seeded in 96-well plate and treated with AMPC, 5-FU, or a combination at constant ratios for 72 h. The specific IC50 of 5-FU and AMPC was determined previously for both Caco2 (5-FU: 2.55 ± 0.4 μM and AMPC: 4.65 ± 0.26 μM) and SW620 (5-FU: 5.12 ± 0.93 μM and AMPC: 2.63 ± 0.69 μM) cell lines. Cell viability was determined by AlamarBlue assay. Fold change in cell viability is plotted. (**D**) Foci formation assay for the drug combination of AMPC and 5-FU in SW620 cells. Cells were seeded at 5000 cells/well in six-well plates and treated with AMPC, 5-FU, or a combination (drug concentrations are 0.25-fold of IC50) for 14 days. The foci were stained with crystal violet. (**E**) Three-dimensional Matrigel growth assay for the drug combination of AMPC and 5-FU in SW620 cells. Cells were grown for three days prior to treatment with AMPC, 5-FU, or a combination at constant ratios. After 14 days, cell viability was measured using AlamarBlue. (**F**) Combination index (CI) plots of AMPC and 5-FU combination treatment in SW620 cells. The solid line represents simulated curve for CI values of fraction affected (Fa) values between 0 to 1. The red dots represent experimental points used to derive the curve. Fa corresponds to the cell killing effect, where 0.0 represents 0% cell death and 1.0 represents 100% cell death. CI < 1.0 indicate synergism. (**G**) Dose reduction index (DRI) plots of AMPC and 5-FU combination treatment in SW620 cells. DRI > 0 indicates favourable combination.

5-Fluorouracil (5-FU) is one component of first line chemotherapeutic regimens, including FOLFIRI and FOLFOX used for the treatment of advanced CRC. However, intrinsic resistance to 5-FU is a major clinical challenge [31]. Based on the COMICS cell line project, a positive correlation trend between TFF3 expression and 5-FU IC_50_ was observed in the CMS4 CRC subtype (Figure S2C). We further examined the effect of TFF3 in decreasing CMS4 CRC cell sensitivity to 5-FU. The IC_50_ values of 5-FU in Caco2-Vec and Caco2-TFF3 cells was determined to be 2.24 ± 0.3 μM and 13.15 ± 2.74 μM respectively (Figure 7A), indicating the reduced sensitivity to 5-FU with forced expression of TFF3. Consistently, the depletion of TFF3 enhanced sensitivity of SW620 cells to 5-FU with a decrease of 5-FU IC_50_ from 6.87 ± 1.93 μM to 3.57 ± 0.32 μM (Figure 7A). Interestingly, an increase in TFF3 expression was observed in response to 5-FU treatment, suggesting the possible involvement of TFF3 as a feedback survival mechanism in 5-FU–based treatment (Figure 7B). Concordant with the increased TFF3 expression, elevated ERK1/2 phosphorylation was also observed upon 5-FU treatment (Figure 7B). This is consistent with the literature where 5-FU was reported to induced both ERK1/2 and AKT activation in oesophageal squamous cell carcinoma [32]. Hence, increased TFF3 expression reduced sensitivity of CMS4 CRC cells to 5-FU.

Drug combinations generally produce improved therapeutic outcomes as compared to single agent treatment and delay the onset of resistance [33]. As such, we explored the combination effects of AMPC and 5-FU in CMS4 CRC cells. The combination resulted in enhanced inhibition of cell viability as compared to either drug alone and as observed by the shift of the combination curve as compared to single treatment curves (Figure 7C and Figure S4A). This is consistent with the observation that TFF3 depletion increased 5-FU sensitivity in SW620 cells (Figure 7A). Moreover, AMPC and 5-FU combination treatment strongly inhibited foci formation in SW620 and Caco2 as compared to single agent treatments (Figure 7D and Figure S4B). Furthermore, as compared to AMPC or 5-FU treatment alone, the combination resulted in enhanced inhibition of SW620 cell growth in 3D Matrigel (Figure 7E).

To examine whether the combination effect between these two drugs was synergistic or additive, Chou-Talalay analysis was performed using Compusyn software. The combination index (CI) values at different fraction affect (Fa) values were calculated based on the median-effect points of the single drugs or drug combination at fixed ratios. The combination of AMPC and 5-FU was observed to be synergistic in SW620 and Caco2 cells with CI values constantly below 1 for Fa values ranging from 0.5 to 0.8 (average CI_0.5-0.8_ = 0.576) (Figure 7F and Figure S4C). The dose reduction index (DRI) for both 5-FU and AMPC were positive (Figure 7G and Figure S4D). In particular, when AMPC was administered in combination, a greater than 10-fold dose reduction of 5-FU was sufficient to achieve similar therapeutic effects in CMS4 CRC cells as compared to when 5-FU was given as a single agent (Figure 7G and Figure S4D). Therefore, the combination of 5-FU and AMPC exerted inhibitory effects on the growth of CMS4 CRC cells in a synergistic manner.

## 3. Discussion

The FOLFIRI or FOLFOX chemotherapy-containing regimens remain the mainstay for the treatment of all stage II/III CRC patients, with no clinical stratification-based treatment in CRC routinely used to date [34]. Previously, anti-EGFR and anti-VEGF targeted therapies, were approved as first line treatments in combination with chemotherapy [35]. However, the anti-EGFR antibody Cetuximab only benefits a subset of CRC patients with wildtype KRAS status and drug resistance and relapse are common in CRC patients who initially respond [36]. Despite the absence of clinical stratification, CRC, like other cancers, is known to exhibit different molecular types, clinical outcome and response to therapies [3,37]. A robust classification of CRC is the CMS subtype, in which CRC is divided into four subtypes (CMS1, CMS2, CMS3, CMS4) based on the consolidation of six independent classification systems in CRC [4]. Several studies have reported the value of CMS subtyping in predicting response to therapies including 5-FU [9,38]. The CMS4 subtype of CRC exhibits the worst prognosis and is associated with chemoresistance [9]. Herein, we demonstrated that TFF3 is a critical driver of progression of the CMS4 subtype of CRC. We have also provided evidence that pharmacological inhibition of TFF3 with a novel specific small molecule TFF3 inhibitor in CMS4 CRC suppresses TFF3-induced oncogenic behaviour and potentiates the efficacy of 5-FU.

Growing evidence supports a role of TFF3 in CRC metastasis and as a predictor of poor prognosis. Increased TFF3 expression has been frequently detected in CRC clinical samples and increased expression of TFF3 was associated with high histological grade, metastasis, and higher TNM stage [39]. An earlier study in an in vivo rat model showed that high expression of TFF3 conferred more aggressive properties to colon cancer cells including enhanced migration and invasion with increased survival [39]. Likewise, TFF3 expression was significantly associated with distant metastasis in CRC patients and resulted in worse overall survival [14,20,40,41]. In contrast, two groups reported protective functions of TFF3 in CRC. It was reported that highly invasive colon tumours were characterized by decreased expression of TFF3 [42]. Furthermore, studies by Uchino et al. reported that TFF3 expression was inversely correlated with CRC progression and that TFF3 suppressed the growth of CRC cells [43,44]. However, they did report that TFF3 enhanced the migratory phenotype of CRC cells in 3D collagen-gel culture [43]. Herein, it was observed that high TFF3 mRNA expression was correlated with worse survival outcome, specifically in the CMS4 subtype. Consistent with previous reports in various cancers [27,31,45], we demonstrated that TFF3 promoted proliferation, survival, oncogenicity, migration and invasion of CMS4 CRC cells, and is required for full growth of CMS4 colorectal tumours in vivo. TFF3 behaves as a promiscuous ligand with resultant activation of multiple l downstream signalling pathways, including AKT and ERK [46,47]. Accordingly, our study has shown that TFF3 activated ERK1/2 in CMS4 CRC cells to mediate cell survival. Furthermore, TFF3 was reported to be upregulated after chemoradiotherapy in CRC, resulting in reduced sensitivity to treatment and higher risk of relapse [21]. Similarly, we report herein that TFF3 expression was increased upon 5-FU treatment and such elevation in TFF3 enhanced CMS4 CRC cell resistance to 5-FU. We also observed that ERK1/2 was activated in the CSC enriched population compared with normal monolayer cultured SW620 cells. These results support the idea that inhibition of TFF3 in combination with conventional chemotherapeutics may be a useful therapeutic approach.

Given that TFF3 is a validated therapeutic target in CMS4 CRC, we explored the use of the first-in-class TFF3 inhibitor in CMS4-CRC. Consistent with siRNA-mediated depletion of TFF3, AMPC inhibition of TFF3 in CMS4 CRC cells resulted in reduction of oncogenic behaviours: cell proliferation, cell survival, foci formation, 3D Matrigel growth, and reduced CSC-like behaviour. As mentioned, TFF3 has been reported to activate the HER family of receptor tyrosine kinases and its associated downstream pathways, including MAPK, AKT, and STAT3 [26]. We also demonstrated that inhibition of TFF3 by AMPC decreased ERK1/2 activation in a dose-dependent manner. In addition to showing efficacy as a single agent, AMPC when used in combination with 5-FU exhibited a synergistic inhibitory effect, which is consistent with our observation that TFF3 depletion increased 5-FU sensitivity in CMS4 CRC cells. Hence, use of AMPC in TFF3 positive CMS4 CRC warrants further consideration.

In conclusion, TFF3 exerts a potent role in promoting CMS4 CRC progression. Thus, TFF3 may function as a potential prognostic biomarker and therapeutic target in CMS4 CRC. As such, the pharmacological inhibition of TFF3 by the novel TFF3 inhibitor AMPC may potentially improve outcomes in the treatment of TFF3-positive CMS4 CRC, either as a single agent or in combination with conventional 5-FU.

## 4. Materials and Methods

### 4.1. Cell Line Maintenance and Cell Transfection

Human colorectal cancer cell lines used in this project were obtained from ATCC (ATCC, Rockville, MD, USA) and were maintained as per recommended instructions from ATCC. SW620 cells and Caco2 cells were obtained from Prof. Richie Soong’s lab and Prof. Yoshiaki Ito’s lab, respectively at the Cancer Science Institute of Singapore (CSI), National University of Singapore (NUS) (Singapore). Cell lines were cultured in Dulbecco’s modified eagle culture media (DMEM) (Nacalai Tesque, Singapore) containing 10% fetal bovine serum (FBS) (Hyclone, Logan, UT) and 1% Penicillin-Streptomycin (Invitrogen, Carlsbad, CA, USA).

Caco2 cells were stably transfected with an expression vector containing full length *TFF3* gene (designated as Caco2-TFF3 cells) or the empty vector (designated as Caco2-vector cells) as previously described [48]. Silencer^®^ Select TFF3 siRNA (Thermo Fisher Scientific, Waltham, MA, USA) used in this project were s277470 and s14041 which were designed to target exon 3 of the TFF3 mRNA. Silencer^®^ Select Negative Control No. 1 siRNA (Thermo Fisher Scientific) was used as the control. The cells were harvested 24 h after transfection for RNA and protein extraction.

### 4.2. Three-Dimensional Matrigel^®^ Growth Culture 

Corning^®^ Matrigel^®^ Matrix, Growth Factor Reduced, Corning^®^ (BD Biosciences, San Diego, CA, USA) was thawed overnight at 4 °C. Black-walled 96-well culture plate was coated with 50 μL 100% Matrigel per well before cells were seeded. Cells were re-suspended in 200 μL media (4% Matrigel and 5% serum) and seeded at a density of 1000 cells per well on the solidified Matrigel base. Drug treatment was performed after 72 h. Drug was replenished at three-day intervals when the media was changed. After culture for 8–10 days, LIVE/DEAD^TM^ Cell Imaging kit (Invitrogen, Carlsbad, CA, USA) was employed to identify live and dead cells using a fluorescence microscope. Cell viability was measured by fluorescence-based readout using AlamarBlue^®^ (Thermo Fisher Scientific).

### 4.3. Annexin V and Propidium Iodide Assay

Phosphatidylserine exposure and cell death were assessed by FACS analysis using Annexin-V-FLUOS Staining Kit (Life Technologies, Gaithersburg, MD, USA) and PI-stained cells as described previously [49]. Briefly, 15–30 × 10^4^ cells were seeded per well in six-well plates. Cells were incubated with serum-free media or drug- containing media to induce apoptosis. After 24–72 h, cells were collected, washed with cold PBS, and re-pelleted. Cells were suspended in 100 μl 1X Annexin binding buffer with addition of 5 μl Annexin V and 1 μL of 100 μg/mL PI. After incubation at room temperature for 15 min, 400 μL of 1X Annexin binding buffer was added and cells were immediately analysed by a BD FACS Aria Cell Sorter (BD Biosciences).

### 4.4. Cell Cycle Assay 

Here, 15–30 × 10^4^ cells were seeded in each well of six-well plates. Cells were harvested and centrifuged for five minutes before resuspension as single cells in 500 μL PBS. Then, 1 mL EtOH (80% in PBS or Milli-Q water) was added into cell suspension with constant vortexing to prevent cells from clustering during fixation. Subsequently, cells were incubated at –20 °C for 15 min for immediate assay or stored.

For assay determination, cells were centrifuged at 1500 rpm for five minutes, resuspended in 2 mL PBS and centrifuged again at 1500 rpm for 5 min. Then, 500 μL PBS solution containing RNaseA (50 μg/mL) and Triton X-100 (1:1000) was added to cell pellets and incubated for 30 min at room temperature. After incubation, PI (10 μg/mL) was added to the solution and gently mixed before further incubation at room temperature for 5 min and flow cytometry analysis.

### 4.5. Caspase-3/7 Activity Assay

Apoptotic cell death was measured by Caspase-Glo^®^ 3/7 assay kit (Promega Madison, WI) according to manufacturer’s instructions. Then, 3 × 10^3^ to 10^4^ cells were seeded in triplicates in white-walled 96-well plates and left to attach overnight. The next day, serum-free media or media with drug were added to cells. After 12–24 h incubation, Caspase-Glo^®^ 3/7 Reagent mixed with media 1:1 was added into each well. The plates were incubated in the dark at room temperature for at least 30 min. Subsequently, apoptosis was determined by measuring caspase-3/7 luminescence activity using the TECAN reader.

### 4.6. Foci Formation Assay 

Here, 10^3^ to 10^4^ cells (in single cell suspension) were plated per well in six-well plates in 2 mL media with or without drug and cultured for 10–14 days. Cells were washed carefully with ice-cold PBS two times and fixed with ice-cold pure methanol for 10 min. After removing methanol, 1 mL crystal violet solution (1% crystal violet in 80 % distilled H_2_O and 20 % methanol) was used to stain cells at room temperature for 10 min. Crystal violet solution was removed and cells were gently rinsed with Milli-Q water to remove the excess crystal violet. The plates were dried at room temperature.

### 4.7. Migration and Invasion Assay

Migration/invasion assays were performed using BD cell culture inserts (BD Biosciences, San Jose, CA, USA) with a pore size of 8 μm on 24-well culture plates. For invasion assays, the inserts were first coated with 10% Matrigel^TM^ diluted in serum-free media. Cells were seeded (1 × 10^4^ cells for migration assay and 2 × 10^4^ cells for invasion assay) with serum-free media in the upper chamber only, while 10% FBS containing media in the bottom well was used as a chemoattractant. After incubation for 24 h, the transwell inserts were washed with PBS and fixed with 4% PFA for 15 min. The non-migrated and non-invaded cells were removed using cotton buds. Migrated or invaded cells on the lower surface of the insert were stained with Hoescht (Sigma-Aldrich, St. Louis, MO, USA) solution (1% triton X 100 and 4 μg/mL Hoechst in PBS) and incubated in the dark at room temperature for 15 min. After rinsing with PBS, cells were counted under the fluorescence microscope.

### 4.8. Colonosphere Formation

Corning^®^ Costar^®^ Ultra-low attachment plates (Corning, Tewksbury, MA, USA) were used for colonosphere culture. The growth media consisted of DMEM-F12 media (Hyclone) supplemented with 2% B27 (Thermo Fisher Scientific, Waltham, MA), 20 ng/mL recombinant human EGF (Sigma-Aldrich), 10 ng/mL recombinant human basic FGF (BD Biosciences), and 5 μg/mL bovine insulin (Sigma-Aldrich). In 96-well plates, 100–200 single cells were seeded in 96 well plates, and the number of colonies in each well was determined after seven days (diameter greater than 60 μm) under the microscope. The colonosphere viability was determined by AlamarBlue^®^ assay (Thermo Fisher Scientific, Waltham, MA). For mRNA and protein extraction, 5000 single cells were seeded in six-well plates and similarly processed.

### 4.9. ALDEFLUOR Assay

The ALDEFLUOR assay was performed using ALDEFLUOR Kit (STEMCELL Technologies, USA) to determine aldehyde dehydrogenase 1 (ALDH1) activity. Cells were seeded in six-well plates. The attached cells were treated with drug or DMSO control in 2% FBS media for 24–48 h. Cells were harvested, washed with PBS and re-suspended in ALDH buffer at a concentration of 10^6^ cells/mL. Subsequently, each 1 mL cell suspension was added to respective ALDH tubes containing 2 μl of ALDH substrate BODIPYTM aminoacetaldehyde (BAAA) and half of the cell suspension was immediately added to the negative control tube containing 1μl of ALDH1 inhibitor diethylaminobenzaldehyde (DEAB) reagent for inactivation of ALDH-catalyzed reaction. Cells were incubated at 37 °C for 25 min and pelleted at 300 rpm for 5 min at 4 °C. Then, 200 μl of supernatant were discarded and replaced by 200 μL fresh ALDH buffer. Cells were kept on ice and processed for subsequent FACS analysis.

### 4.10. Polymerase Chain Reaction (PCR) and Western Blot Analysis

Semiquantitative RT-PCR and quantitative polymerase chain reaction (PCR) were performed as previously described [50]. Western blot analysis was performed using the following antibodies: rabbit TFF3 monoclonal antibody (Abcam, ab108599), mouse β-ACTIN monoclonal antibody (Santa Cruz, sc-47778), rabbit ERK1/2 monoclonal antibody (Cell Signalling, 4695S), rabbit p-ERK1/2 monoclonal antibody (Cell Signalling, 9101S), goat ALDH1A1 monoclonal antibody (Santa Cruz, sc-22589), rabbit p-JNK (T183/Y185) antibody (Cell Signalling, 9251S), rabbit JNK antibody (Cell Signalling, 9252S), rabbit p-p38 (T180/Y182) antibody (Cell Signalling, 9211S), and rabbit p38 antibody (Cell Signalling, 9212S). Before blotting some membranes were cut into multiple portions dependent on molecular weight to facilitate the detection of multiple proteins.

### 4.11. Animal Care

Four- to five-week-old male BALB/c athymic nude mice were acquired from the Vital River Laboratory Animal Technology Co., Ltd. (Beijing, China) and acclimated for one week. All animals were housed in a controlled atmosphere (25 ± 1 °C at 50% relative humidity) under a 12-h light/12-h dark cycle. Animals had free access to food and water at all times. Food cups were replenished with fresh diet every day. The utilized animal experimental protocols received approved from both the Institutional Animal Care and Use Committee of the Laboratory Animal Centre of Peking University Shenzhen and the Tsinghua Shenzhen Graduate School (the permit from Peking University Shenzhen Graduate School is “YW”: the permit from Tsinghua Shenzhen International Graduate School is “ethical development no. 37 (Year 2019)”.

### 4.12. Subcutaneous SW620 Xenograft Tumour Model

BALB/c athymic nude mice were subcutaneously injected with SW620 cells (5 × 10^6^ in 0.2 mL of PBS) in the right flank. Five days later (tumour volume had reached approximately 100 mm^3^), the mice were randomly divided into two groups (*n* = 8) and intraperitoneally injected daily with AMPC at a dose of 40 mg/kg body weight or equivalent amount of vehicle for two weeks (summarized in Figure 5A). All mice received vehicle in volumes equivalent to those used for injection of AMPC. Animal body weight and tumour size were measured and recorded every day using an electronic balance and a digital square, respectively. Tumour volume (mm^3^) was calculated using the formula: 0.52 × length ×[width]^2^. All mice were sacrificed at 21 days, and the tumour masses were dissected. The dissected tumours were placed in 10% buffered formalin, embedded in paraffin and cut in 4 μm sections for hematoxylin and eosin (H&E) and immunohistochemistry (IHC).

### 4.13. Measurement of TFF3 Levels

Mouse serum was collected, centrifuged and stored at –80 °C prior to testing. Human TFF3 levels in the mouse serum were quantified by colorimetric determination using a specific human TFF3 Quantikine ELISA kit (R&D systems, Minneapolis, MN) according to the manufacturer’s instructions. The serum samples were not reduced prior to ELISA. For clarity, it should be noted that the ELISA assay may exhibit differences in immunoreactivity between TFF3 monomer/dimer and/or other potential heterodimers with TFF3.

### 4.14. Statistical Analysis

Data was expressed as mean ± SD and statistical analysis was performed using unpaired Student’s t-test. A p-value of less than 0.05 was considered statistically significant. The graphical presentations were prepared using GraphPad Prism 5 (GraphPad Software, Inc., CA, USA). Drug combination index was calculated by the Compusyn software (Dr. Dorothy Chou) using a constant drug concentration ratio.

## 5. Conclusions

The present study provides evidence for CMS subtype-based intervention in CRC. With TFF3 expression being correlated with poor prognosis in the CMS4 subtype of CRC, we demonstrated that TFF3 promotes oncogenic and CSC-like behaviour of CMS4 CRC cells. The pharmacological inhibition of TFF3 by a novel small molecule inhibitor, AMPC, significantly decreased the oncogenic behaviour of CMS4 CRC cells. Furthermore, TFF3 decreased the sensitivity of CMS4 CRC cells to 5-FU. AMPC when used in combination with 5-FU exhibited a synergistic effect in CMS4 CRC cells. In summary, this study highlights the potential of TFF3 as a therapeutic target in CMS4 CRC and provides preclinical evidence that inhibition of TFF3 with AMPC may be useful in this subtype of CRC.

## 6. Patents

V.P., B., and P.E.L. are named as inventors on PCT application number WO/2018/226155, Compounds, As Inhibitors of TFF3 Dimerization, Methods and Applications Thereof [19].

## Supplementary Materials

Supplementary materials can be found at https://www.mdpi.com/1422-0067/20/24/6215/s1. Figure S1. Clinical relevance of TFF3 in CRC. Figure S2: TFF3 expression in CMS4 CRC cell lines. Figure S3. Functional effects of inhibition of TFF3 by AMPC in Caco2 cells. Figure S4. Synergistic inhibitory effects of AMPC and 5-FU in Caco2 cells.

## References

[1] Manfredi S., Lepage C., Hatem C., Coatmeur O., Faivre J., Bouvier A.M. (2006). Epidemiology and management of liver metastases from colorectal cancer. Ann. Surg..

[2] Nordlinger B., Van Cutsem E., Rougier P., Köhne C.H., Ychou M., Sobrero A., Adam R., Arvidsson D., Carrato A., Georgoulias V. (2007). Does chemotherapy prior to liver resection increase the potential for cure in patients with metastatic colorectal cancer? A report from the European Colorectal Metastases Treatment Group. Eur. J. Cancer.

[3] Pal R., Wei N., Song N., Wu S., Kim R.S., Wang Y., Gavin P.G., Lucas P.C., Srinivasan A., Allegra C.J. (2018). Molecular subtypes of colorectal cancer in pre-clinical models show differential response to targeted therapies: Treatment implications beyond KRAS mutations. PLoS ONE.

[4] Guinney J., Dienstmann R., Wang X., de Reyniès A., Schlicker A., Soneson C., Marisa L., Roepman P., Nyamundanda G., Angelino P. (2015). The consensus molecular subtypes of colorectal cancer. Nat. Med..

[5] Müller M.F., Ibrahim A.E., Arends M.J. (2016). Molecular pathological classification of colorectal cancer. Virchows Arch..

[6] Song N., Pogue-Geile K.L., Gavin P.G., Yothers G., Kim S.R., Johnson N.L., Lipchik C., Allegra C.J., Petrelli N.J., O’Connell M.J. (2016). Clinical outcome from oxaliplatin treatment in stage II/III colon cancer according to intrinsic subtypes: Secondary analysis of NSABP C-07/NRG oncology randomized clinical trial. JAMA Oncol..

[7] Del Rio M., Mollevi C., Bibeau F., Vie N., Selves J., Emile J.F., Roger P., Gongora C., Robert J., Tubiana-Mathieu N. (2017). Molecular subtypes of metastatic colorectal cancer are associated with patient response to irinotecan-based therapies. Eur. J. Cancer.

[8] Fessler E., Medema J.P. (2016). Colorectal cancer subtypes: Developmental origin and microenvironmental regulation. Trends Cancer.

[9] Linnekamp J.F., Hooff S.R.V., Prasetyanti P.R., Kandimalla R., Buikhuisen J.Y., Fessler E., Ramesh P., Lee K.A.S.T., Bochove G.G.W., de Jong J.H. (2018). Consensus molecular subtypes of colorectal cancer are recapitulated in in vitro and in vivo models. Cell Death Differ..

[10] Williams G., Wright N. (1997). Trefoil factor family domain peptides. Virchows Arch..

[11] Thim L. (1997). Trefoil peptides: From structure to function. Cell. Mol. Life Sci..

[12] Aihara E., Engevik K.A., Montrose M.H. (2017). Trefoil factor peptides and gastrointestinal function. Annu. Rev. Physiol..

[13] Xue H., Lü B., Zhang J., Wu M., Huang Q., Wu Q., Sheng H., Wu D., Hu J., Lai M. (2009). Identification of serum biomarkers for colorectal cancer metastasis using a differential secretome approach. J. Proteome Res..

[14] Babyatsky M., Lin J., Yio X., Chen A., Zhang J.Y., Zheng Y., Twyman C., Bao X., Schwartz M., Thung S. (2009). Trefoil factor-3 expression in human colon cancer liver metastasis. Clin. Exp. Metastasis.

[15] Thim L., May F.E. (2005). Structure of mammalian trefoil factors and functional insights. Cell. Mol. Life Sci..

[16] Albert T.K., Laubinger W., Müller S., Hanisch F.G., Kalinski T., Meyer F., Hoffmann W. (2010). Human intestinal TFF3 forms disulfide-linked heteromers with the mucus-associated FCGBP protein and is released by hydrogen sulfide. J. Proteome Res..

[17] Madsen J., Sorensen G.L., Nielsen O., Tornøe I., Thim L., Fenger C., Mollenhauer J., Holmskov U. (2013). A variant form of the human deleted in malignant brain tumor 1 (DMBT1) gene shows increased expression in inflammatory bowel diseases and interacts with dimeric trefoil factor 3 (TFF3). PLoS ONE.

[18] Poulsen S.S., Kissow H., Hare K., Hartmann B., Thim L. (2005). Luminal and parenteral TFF2 and TFF3 dimer and monomer in two models of experimental colitis in the rat. Regul. Pept..

[19] Lobie P.E., Pandey V., Kanchugarakoppal S.R., Basappa Chakrabhavi D.M., Rangappa S. (2018). Compounds Useful in Inhibiting Human Trefoil Factor 3. Patent.

[20] Morito K., Nakamura J., Kitajima Y., Kai K., Tanaka T., Kubo H., Miyake S., Noshiro H. (2015). The value of trefoil factor 3 expression in predicting the long-term outcome and early recurrence of colorectal cancer. Int. J. Oncol..

[21] Casado E., Garcia V.M., Sánchez J.J., Gómez Del Pulgar M.T., Feliu J., Maurel J., Castelo B., Moreno Rubio J., López R.A., García-Cabezas M.Á. (2012). Upregulation of trefoil factor 3 (TFF3) after rectal cancer chemoradiotherapy is an adverse prognostic factor and a potential therapeutic target. Int. J. Radiat. Oncol. Biol. Phys..

[22] Kubens B., Zänker K.S. (1998). Differences in the migration capacity of primary human colon carcinoma cells (SW480) and their lymph node metastatic derivatives (SW620). Cancer Lett..

[23] Abdullah L.N., Chow E.K.-H. (2013). Mechanisms of chemoresistance in cancer stem cells. Clin. Transl. Med..

[24] Ahmed N., Abubaker K., Findlay J., Quinn M. (2010). Epithelial mesenchymal transition and cancer stem cell-like phenotypes facilitate chemoresistance in recurrent ovarian cancer. Curr. Cancer Drug Targets.

[25] Shaheen S., Ahmed M., Lorenzi F., Nateri A.S. (2016). Spheroid-formation (colonosphere) assay for in vitro assessment and expansion of stem cells in colon cancer. Stem Cell Rev. Rep..

[26] Chong Q.-Y., You M.L., Pandey V., Banerjee A., Chen Y.J., Poh H.M., Zhang M., Ma L., Zhu T., Basappa S. (2017). Release of HER2 repression of trefoil factor 3 (TFF3) expression mediates trastuzumab resistance in HER2+/ER+ mammary carcinoma. Oncotarget.

[27] You M.-L., Chen Y.J., Chong Q.Y., Wu M.M., Pandey V., Chen R.M., Liu L., Ma L., Wu Z.S., Zhu T. (2017). Trefoil factor 3 mediation of oncogenicity and chemoresistance in hepatocellular carcinoma is AKT-BCL-2 dependent. Oncotarget.

[28] Douville J., Beaulieu R., Balicki D. (2009). ALDH1 as a functional marker of cancer stem and progenitor cells. Stem Cells Dev..

[29] Kannan N., Kang J., Kong X., Tang J., Perry J.K., Mohankumar K.M., Miller L.D., Liu E.T., Mertani H.C., Zhu T. (2010). Trefoil factor 3 is oncogenic and mediates anti-estrogen resistance in human mammary carcinoma. Neoplasia.

[30] Kinoshita K., Taupin D.R., Itoh H., Podolsky D.K. (2000). Distinct pathways of cell migration and antiapoptotic response to epithelial injury: Structure-function analysis of human intestinal trefoil factor. Mol. Cell. Biol..

[31] Hammond W.A., Swaika A., Mody K. (2016). Pharmacologic resistance in colorectal cancer: A review. Ther. Adv. Med. Oncol..

[32] Lu Y.-X., Chen D.L., Wang D.S., Chen L.Z., Mo H.Y., Sheng H., Bai L., Wu Q.N., Yu H.E., Xie D. (2016). Melatonin enhances sensitivity to fluorouracil in oesophageal squamous cell carcinoma through inhibition of Erk and Akt pathway. Cell Death Dis..

[33] Zanardi E., Bregni G., De Braud F., Di Cosimo S. (2015). Better together: Targeted combination therapies in breast cancer. Seminars in Oncology.

[34] Mohelnikova-Duchonova B., Melichar B., Soucek P. (2014). FOLFOX/FOLFIRI pharmacogenetics: The call for a personalized approach in colorectal cancer therapy. World J. Gastroenterol..

[35] Seeber A., Gastl G. (2016). Targeted Therapy of Colorectal Cancer. Oncol. Res. Treat..

[36] Misale S., Di Nicolantonio F., Sartore-Bianchi A., Siena S., Bardelli A. (2014). Resistance to anti-EGFR therapy in colorectal cancer: From heterogeneity to convergent evolution. Cancer Discov..

[37] Bae J.M., Kim J.H., Kang G.H. (2016). Molecular subtypes of colorectal cancer and their clinicopathologic features, with an emphasis on the serrated neoplasia pathway. Arch. Pathol. Lab. Med..

[38] Thanki K., Nicholls M.E., Gajjar A., Senagore A.J., Qiu S., Szabo C., Hellmich M.R., Chao C. (2017). Consensus Molecular Subtypes of Colorectal Cancer and their Clinical Implications. Int. Biol. Biomed. J..

[39] Yio X., Zhang J.Y., Babyatsky M., Chen A., Lin J., Fan Q.X., Werther J.L., Itzkowitz S. (2005). Trefoil factor family-3 is associated with aggressive behavior of colon cancer cells. Clin. Exp. Metastasis.

[40] Vocka M., Langer D., Petrtyl J., Vockova P., Hanus T., Kalousova M., Zima T., Petruzelka L. (2015). Trefoil factor family (TFF) proteins as potential serum biomarkers in patients with metastatic colorectal cancer. Neoplasma.

[41] Huang Y.-G., Li Y.F., Wang L.P., Zhang Y. (2013). Aberrant expression of trefoil factor 3 is associated with colorectal carcinoma metastasis. J. Cancer Res. Ther..

[42] John R., El-Rouby N.M., Tomasetto C., Rio M.C., Karam S.M. (2007). Expression of TFF3 during multistep colon carcinogenesis. Histol. Histopathol..

[43] Uchino H., Kataoka H., Itoh H., Hamasuna R., Koono M. (2000). Overexpression of intestinal trefoil factor in human colon carcinoma cells reduces cellular growth in vitro and in vivo. Gastroenterology.

[44] Uchino H., Kataoka H., Itoh H., Koono M. (1997). Expression of intestinal trefoil factor mRNA is downregulated during progression of colorectal carcinomas. J. Clin. Pathol..

[45] Perera O., Evans A., Pertziger M., MacDonald C., Chen H., Liu D.X., Lobie P.E., Perry J.K. (2015). Trefoil factor 3 (TFF3) enhances the oncogenic characteristics of prostate carcinoma cells and reduces sensitivity to ionising radiation. Cancer Lett..

[46] Shi H.-S., Zhu W.L., Liu J.F., Luo Y.X., Si J.J., Wang S.J., Xue Y.X., Ding Z.B., Shi J., Lu L. (2012). PI3K/Akt signaling pathway in the basolateral amygdala mediates the rapid antidepressant-like effects of trefoil factor 3. Neuropsychopharmacology.

[47] Baus-Loncar M., Giraud A.S. (2005). Multiple regulatory pathways for trefoil factor (TFF) genes. Cell. Mol. Life Sci..

[48] Pandey V., Wu Z.S., Zhang M., Li R., Zhang J., Zhu T., Lobie P.E. (2014). Trefoil factor 3 promotes metastatic seeding and predicts poor survival outcome of patients with mammary carcinoma. Breast Cancer Res..

[49] Keerthy H.K., Garg M., Mohan C.D., Madan V., Kanojia D., Shobith R., Nanjundaswamy S., Mason D.J., Bender A., Basappa (2014). Synthesis and characterization of novel 2-amino-chromene-nitriles that target Bcl-2 in acute myeloid leukemia cell lines. PLoS ONE.

[50] Pandey V., Perry J.K., Mohankumar K.M., Kong X.J., Liu S.M., Wu Z.S., Mitchell M.D., Zhu T., Lobie P.E. (2008). Autocrine human growth hormone stimulates oncogenicity of endometrial carcinoma cells. Endocrinology.

