# Supplementary material for: Pharmacological Inhibition of TFF3 Enhances Sensitivity of CMS4 Colorectal Carcinoma to 5-Fluorouracil through Inhibition of p44/42 MAPK
Source: Int J Mol Sci. 2019 Dec 9;20(24):6215. doi: 10.3390/ijms20246215 (PMC6940926; doi:10.3390/ijms20246215)
Supplement: Supplementary file 1 [file ijms-20-06215-s001.pdf]

Supplementary Figure 1

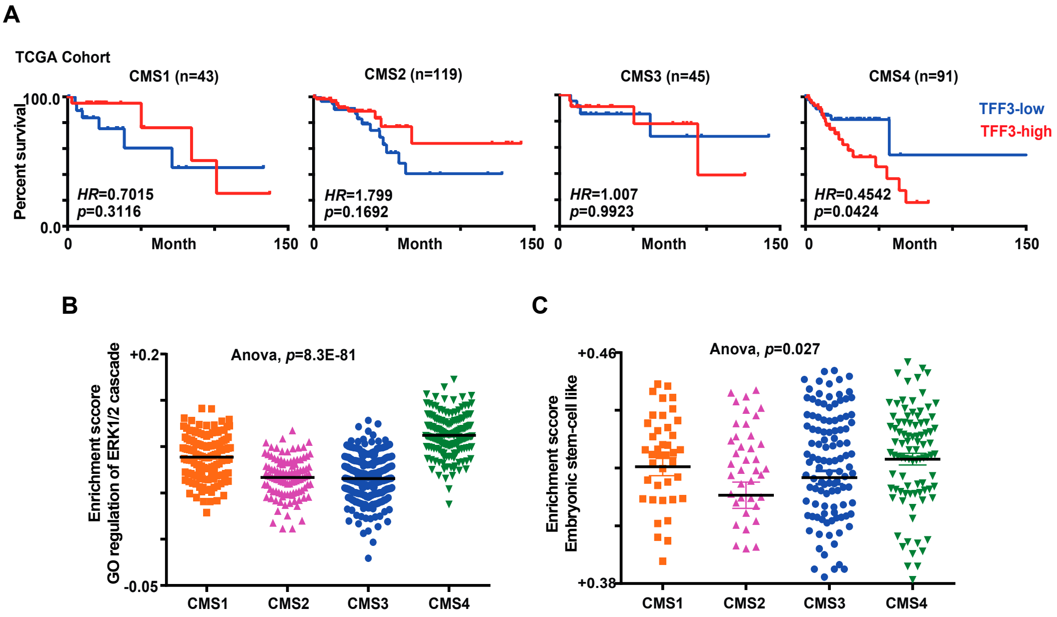

Figure S1. Clinical relevance of TFF3 in CRC.

Supplementary Figure 2

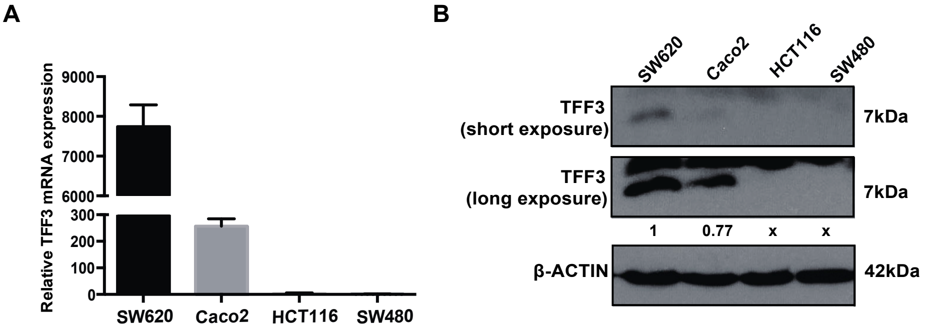

C

Correlation between TFF3 expression and 5-FU IC_{50}

TFF3 vs 5-FU IC_{50}	n	Spearman Rho	Spearman p-value
CMS1	6	-0.0857	0.9194
CMS2	3	-0.5268	0.6468
CMS3	2	---	---
CMS4	6	0.3143	0.5639

Figure S2. TFF3 expression in CMS4 CRC cell lines.

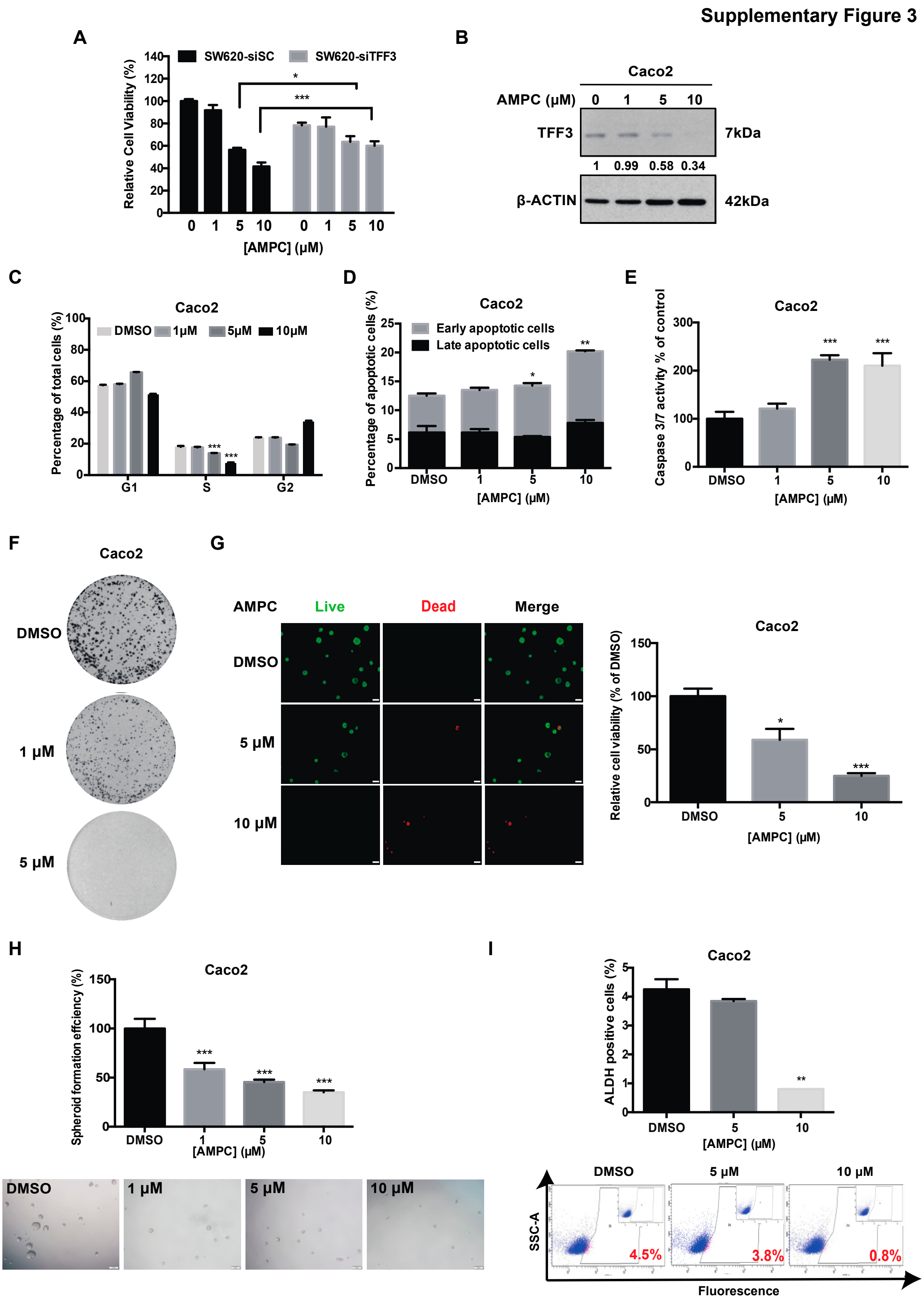

Figure S3. Functional effects of inhibition of TFF3 by AMPC in Caco2 cells.

Supplementary Figure 4

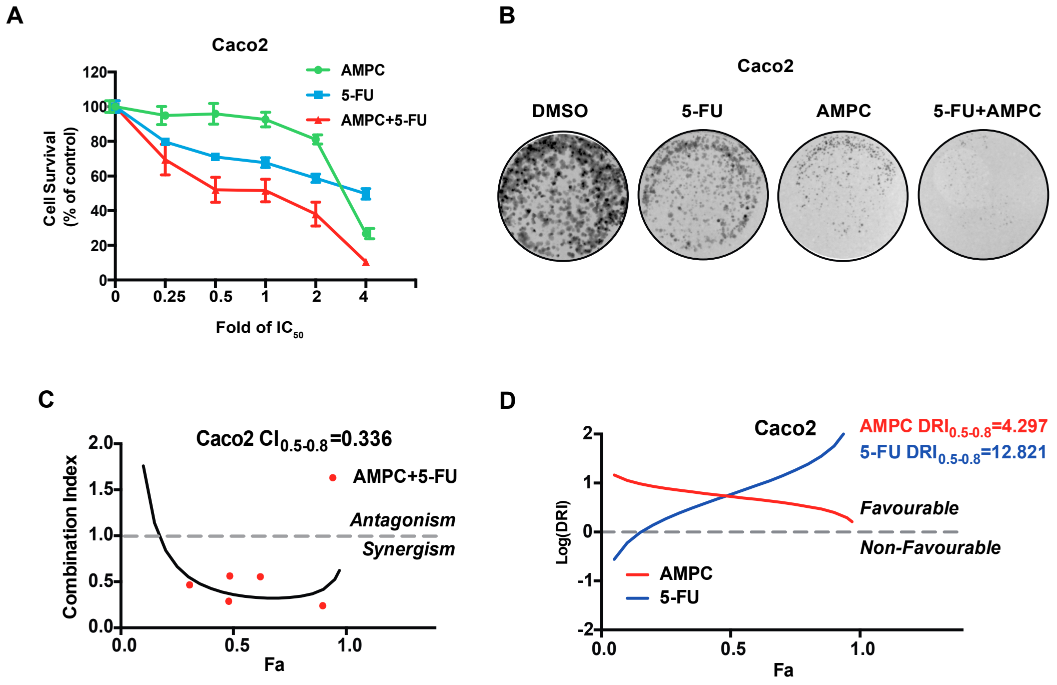

Figure S4. Synergistic inhibitory effects of AMPC and 5-FU in Caco2 cells.
